# Hole‐Activated Lattice Oxygen Enables Pt‐Free Propane Dehydrogenation by Eliminating the Hydrogen Recombination Bottleneck

**DOI:** 10.1002/anie.1329092

**Published:** 2026-07-30

**Authors:** DongHwan Oh, Jaewoo Jeong, Susung Lee, Younghwan Park, Seunghyuck Chi, Yaejun Baik, Hyungjun Kim, Hyeyoung Shin, Minkee Choi

**Affiliations:** ^1^ Department of Chemical and Biomolecular Engineering (BK21 Four) Korea Advanced Institute of Science and Technology (KAIST) Daejeon Republic of Korea; ^2^ Department of Chemistry Korea Advanced Institute of Science and Technology (KAIST) Daejeon Republic of Korea; ^3^ Department of Chemistry, College of Science Kyung Hee University Seoul Republic of Korea

**Keywords:** gallia–alumina, hole species, hydrogen recombination, lattice oxygen, propane dehydrogenation

## Abstract

Gallia–alumina (Ga*_x_* Al_2−_ *_x_* O_3_) enables efficient propane dehydrogenation (PDH) via C–H activation at Ga–O pairs, but the overall rate is limited by sluggish hydrogen recombination to H_2_. Although Pt promotion alleviates this bottleneck, it introduces drawbacks including precious‐metal cost and regeneration‐induced sintering. Here, we show that Mg doping into Ga*_x_* Al_2−_ *_x_* O_3_ electronically activates lattice oxygen to enable rapid H_2_ evolution directly on the oxide, eliminating the need for Pt promotion. Electron paramagnetic resonance spectroscopy reveals the formation of hole‐type oxygen species upon Mg incorporation, while kinetic/isotopic measurements and theoretical calculations demonstrate substantially accelerated hydrogen recombination kinetics. In situ infrared spectroscopy further shows rapid depletion of surface hydrogen species, consistent with facilitated H_2_ formation from Ga–H and O–H intermediates. At 853 K, Mg‐doped Ga*_x_* Al_2−_ *_x_* O_3_ exhibits a threefold higher propylene formation rate than the undoped material at 97% propylene selectivity, outperforming benchmark PtSn/*γ*‐Al_2_O_3_ and CrO*_x_*/*γ*‐Al_2_O_3_ catalysts. Furthermore, the fully oxide‐based catalyst shows the slowest deactivation over 30 dehydrogenation–regeneration cycles. These findings establish lattice‐oxygen electronic engineering as a practical strategy for achieving both high activity and exceptional regeneration stability in oxide‐catalyzed dehydrogenation chemistry.

## Graphical abstract

Trace Mg doping into gallia–alumina generates hole‐type oxygen species that accelerate hydrogen recombination, eliminating the need for Pt promotion. The fully oxide‐based catalyst resists sintering under repeated regeneration, outperforming benchmark PtSn and CrO*_x_* catalysts with exceptional stability over 30 dehydrogenation–regeneration cycles.

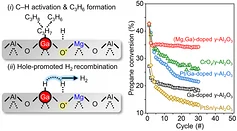

## 1 Introduction

Propylene is a primary feedstock for polypropylene, propylene oxide, acrylic acid, and other bulk chemicals [1, 2]. Historically, a substantial fraction of propylene has been supplied as a co‐product from naphtha steam cracking. The shale‐gas‐driven shift of steam crackers toward ethane‐rich feeds has structurally reduced propylene co‐production, motivating development of on‐purpose technologies such as propane dehydrogenation (PDH) [3, 4, 5]. PDH is highly endothermic and equilibrium‐limited, typically requiring operation at 823–973 K to achieve practical yields [1, 2]. These harsh conditions promote coke formation and catalyst deactivation. Periodic oxidative regeneration of catalysts is thus integral to commercial PDH operation, and the heat released during coke combustion is often deliberately used to offset the endothermic heat demand [1, 2, 6]. For this reason, catalyst development must prioritize not only activity and olefin selectivity but also stability under repeated reaction–regeneration cycling. Notably, while many studies assess catalyst stability within a single dehydrogenation run, regeneration stability is more decisive under industrially relevant cyclic operation.

Commercial PDH catalysts are dominated by Pt‐based bimetallic systems, particularly PtSn/*γ*‐Al_2_O_3_ catalysts, which deliver high propylene selectivity and industrially relevant reaction rates [7, 8, 9]. Beyond PtSn, a variety of Pt‐based alloys and intermetallic catalysts have also been explored to optimize catalytic activity, selectivity, and coke resistance [10, 11, 12, 13, 14]. Nevertheless, despite their high catalytic performance, Pt‐based catalysts remain susceptible to structural evolution under repeated oxidative regeneration. Under cyclic operation, such catalysts may undergo particle sintering and changes in bimetallic structure, including dealloying and promoter redistribution, leading to gradual performance deterioration [8, 9]. To mitigate these issues, PtSn/*γ*‐Al_2_O_3_‐based PDH processes (Oleflex process, Honeywell UOP) often incorporate controlled oxychlorination to maintain catalyst dispersion and performance, adding operational complexity and materials constraints for reactor design [1, 2, 9, 15]. These limitations continue to motivate the development of alternative catalyst systems that retain high catalytic performance while exhibiting greater robustness under repeated dehydrogenation–regeneration cycling.

Oxide catalysts have emerged as attractive alternatives because they can, in principle, provide greater thermal robustness and regeneration tolerance than supported noble‐metal catalysts. CrO*_x_*/*γ*‐Al_2_O_3_ catalysts are widely implemented industrially in the CATOFIN process (Lummus Technology), owing to their superior stability under repeated oxidative regeneration conditions [1, 2, 6]. Nevertheless, environmental and regulatory concerns associated with chromium continue to drive the development of chromium‐free oxide catalysts for PDH [1, 16]. Beyond chromium oxides, a variety of oxide catalyst systems have recently been explored for PDH [2, 17, 18, 19]. Among them, gallia‐based catalysts, including Ga_2_O_3_ and gallia–alumina (Ga*_x_* Al_2−_ *_x_* O_3_), have emerged as promising candidates because Ga–O motifs enable efficient C–H activation while maintaining high propylene selectivity [20, 21, 22, 23, 24, 25]. However, these catalysts still exhibit limited activity because hydrogen recombination to H_2_ remains kinetically sluggish on oxide surfaces, resulting in the accumulation of Ga–H and O–H surface intermediates [26, 27, 28, 29].

Previous studies addressed the limitations of gallia‐based catalysts through trace Pt promotion (<0.1 wt%), creating bifunctional systems in which the oxide mediates C–H activation while Pt accelerates H_2_ formation [27, 28, 29, 30]. This approach synergistically combines the high selectivity and regeneration stability of oxide catalysts with the rapid hydrogen recombination kinetics of Pt, substantially enhancing PDH activity. Nevertheless, Pt promotion does not fully overcome practical limitations, because Pt can still undergo significant sintering during repeated oxidative regeneration [28, 29, 30], while reliance on precious metals raises cost and supply concerns. These challenges motivate the development of a precious‐metal‐free, fully oxide‐based catalyst that accelerates hydrogen recombination directly on the oxide surface while preserving the intrinsic C–H activation capability and regeneration robustness of Ga–O motifs.

In this study, we show that trace Mg doping into Ga*_x_* Al_2−_ *_x_* O_3_ electronically activates lattice oxygen and enables rapid hydrogen recombination directly on the oxide surface without the need for Pt promotion. Electron paramagnetic resonance spectroscopy provides evidence for the formation of hole‐type oxygen species upon Mg incorporation, while kinetic and isotopic measurements, together with theoretical calculations, reveal substantially accelerated hydrogen recombination kinetics. By embedding the hydrogen‐recombination function within a thermally robust oxide lattice, this strategy simultaneously delivers high catalytic activity and exceptional stability under repeated dehydrogenation–regeneration cycling, positioning Mg‐doped Ga*_x_* Al_2−_ *_x_* O_3_ as a promising Pt‐ and Cr‐free catalyst platform for propane dehydrogenation.

## 2 Results and Discussion

### 2.1 Structural Characterization of Mg‐Doped Ga_x_Al_2−x_O_3_ Catalysts

Mg‐doped Ga*_x_* Al_2−_ *_x_* O_3_ catalysts were prepared by impregnating *γ*‐Al_2_O_3_ with aqueous solutions of Mg(NO_3_)_2_ and Ga(NO_3_)_3_, followed by calcination. The Ga loading was fixed at 1 wt%, while the Mg loading was varied from 0.1 to 5 wt%. The catalysts are denoted as “*x*Mg1GaAl,” where *x* represents the Mg loading (wt%). For comparison, a Mg‐free reference catalyst (“1GaAl”) and a 1 wt% Ga/MgAl_2_O_4_ reference catalyst (“1GaMAO”) were additionally prepared. The Mg and Ga contents determined by inductively coupled plasma optical emission spectroscopy (ICP‐OES) are summarized in Table 1, showing that the actual Mg and Ga contents deviated by less than 10% from the nominal values. Surface composition analysis by XPS revealed substantially higher apparent Ga and Mg contents than the bulk values determined by ICP‐OES for 1GaAl and xMg1GaAl catalysts, suggesting surface enrichment of both Ga and Mg species.

**TABLE 1:** Structural properties and chemical compositions of Mg‐doped Ga*_x_* Al_2−_ *_x_* O_3_ catalysts.

Sample	*S*_BET_ ^a^ (m^2^ g^−1^)	*V*_total_ ^b^ (cm^3^ g^−1^)	*d*_NLDFT_ ^c^ (nm)	Ga content^d^ (wt%)	Mg content^d^ (wt%)	Ga surface content^e^ (wt%)	Mg surface content^e^ (wt%)	Ga_IV, surf_ ^f^ (mmol g_cat_^−1^)
1GaAl	217	0.51	9.1	1.05	—	1.71	—	0.068
0.1Mg1GaAl	225	0.52	9.2	1.04	0.10	1.69	0.21	0.059
0.5Mg1GaAl	236	0.52	8.9	1.02	0.50	1.72	1.29	0.056
1.0Mg1GaAl	232	0.52	8.8	1.09	1.06	1.73	2.17	0.051
2.0Mg1GaAl	213	0.51	8.9	1.08	2.06	1.66	2.92	0.041
5.0Mg1GaAl	179	0.38	8.6	1.10	5.04	1.66	5.32	0.016
1GaMAO	160	0.33	8.4	1.03	16.5	1.70	16.3	0.004

^a^ Brunauer–Emmett–Teller (BET) surface area was determined in the *P*/*P*_0_ range of 0.05–0.15.

^b^ Total pore volume was calculated at *P*/*P*_0_ = 0.95.

^c^ Pore size was calculated using the nonlocal density functional theory (NLDFT) analysis.

^d^ Ga and Mg contents were determined by ICP‐OES.

^e^ Ga and Mg surface compositions were quantified by XPS.

^f^ Ga_IV, surf_ sites were determined by propylene temperature‐programmed desorption.

In all Ga‐containing samples, the Ga 2*p*_3/2_ peak in the XPS spectra shifts to higher binding energy (1118.1–1118.2 eV) than that of bulk *β*‐Ga_2_O_3_ (1117.6 eV), indicating the incorporation of Ga into an oxide environment distinct from segregated Ga_2_O_3_ domains (Figure S1) [31, 32, 33]. In temperature‐programmed reduction (TPR) experiments (Figure S2), all Ga‐containing catalysts exhibited no appreciable reduction features over the investigated temperature range, whereas a physical mixture of 1.0 wt% *β*‐Ga_2_O_3_ and *γ*‐Al_2_O_3_ presented a broad H_2_‐consumption feature at around 600 K, which can be associated with hydrogen‐induced partial reduction and defect formation in *β*‐Ga_2_O_3_ [34]. This comparison indicates that Ga^3+^ in all catalysts is not present as segregated Ga_2_O_3_ domains but is instead incorporated into the oxide lattice, forming a non‐reducible solid solution.

The X‐ray diffraction (XRD) patterns of 1GaAl and *x*Mg1GaAl samples with low Mg loadings (*x* ≤0.5) exhibit broad reflections characteristic of defective‐spinel *γ*‐Al_2_O_3_ [35], with no detectable reflections corresponding to secondary crystalline phases (Figure 1a). Scanning electron microscopy (SEM) images reveal that the rod‐like morphology characteristic of *γ*‐Al_2_O_3_ is preserved at low Mg loadings (Figures 1b and S3). Upon increasing the Mg loading above 0.5 wt%, the diffraction features become progressively more characteristic of spinel MgAl_2_O_4_, with enhanced reflections appearing near 2*θ* ≈ 31.3°, 36.8°, and 59.3° corresponding to the (220), (311), and (511) planes, respectively (Figure 1a). This indicates the onset of phase separation at higher Mg contents. Correspondingly, SEM images (Figures 1b and S3) reveal the coexistence of two distinct crystallite morphologies for samples with Mg loadings >0.5 wt%: the original rod‐like crystallites associated with the *γ*‐Al_2_O_3_‐based phase and newly formed plate‐like crystallites characteristic of spinel MgAl_2_O_4_. The relative fraction of the plate‐like crystallites increases with increasing Mg loading above 0.5 wt%.

**FIGURE 1:**
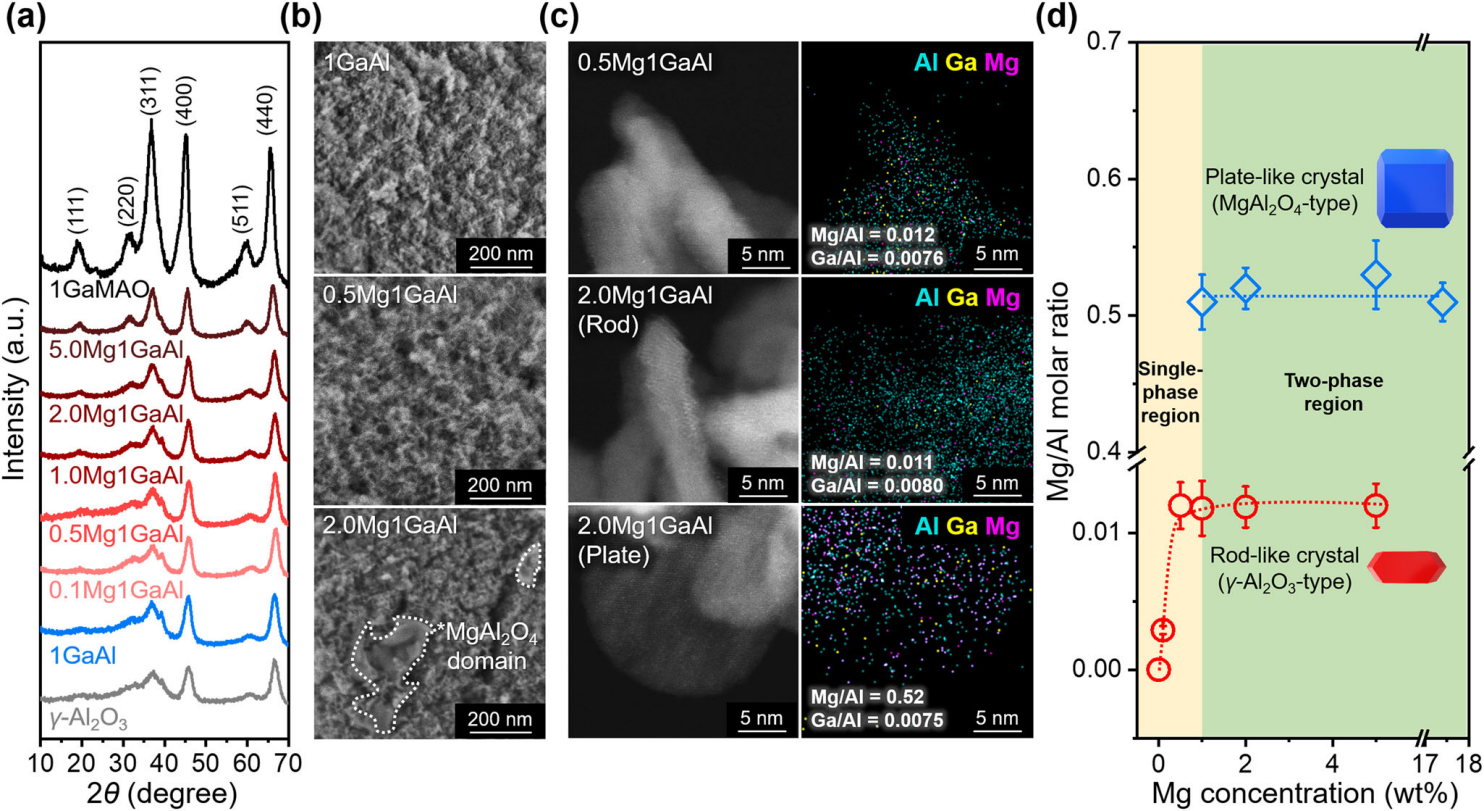
(a) XRD patterns of the prepared catalysts. (b) SEM images of 1GaAl, 0.5Mg1GaAl, and 2.0Mg1GaAl; dotted regions indicate MgAl_2_O_4_ spinel domains. (c) HAADF‐STEM images, corresponding EDS elemental maps, and quantitative elemental compositions for 0.5Mg1GaAl and 2.0Mg1GaAl. The 0.5Mg1GaAl sample exhibits only rod‐like crystal domains with uniform elemental distributions, whereas 2.0Mg1GaAl exhibits both rod‐like and plate‐like domains with distinct elemental compositions. Elemental compositions were quantified by averaging over five independent crystal domains. (d) Average Mg/Al molar ratios for rod‐like and plate‐like crystal domains determined by EDS.

High‐angle annular dark‐field scanning transmission electron microscopy (HAADF‐STEM) images and energy‐dispersive x‐ray spectroscopy (EDS) analysis (Figures 1c and S4) further confirm that *x*Mg1GaAl samples with *x* ≤0.5 consist exclusively of rod‐like crystallites, within which the Mg/Al molar ratio gradually increases with increasing *x* (Figure 1d). These samples exhibit a uniform distribution of both Mg and Ga throughout the oxide crystallites within the spatial resolution of the measurement, suggesting that Mg and Ga are homogeneously incorporated into the *γ*‐Al_2_O_3_ structure. However, when *x* exceeds 0.5, the Mg/Al ratio within the rod‐like domains reaches a plateau value (∼0.012, Figure 1d), while the fraction of plate‐like crystallites continuously increases. The average Mg/Al ratio of the plate‐like crystallites remains approximately 0.52 regardless of the Mg loading, consistent with the stoichiometry of MgAl_2_O_4_ spinel. These observations suggest that Mg exhibits limited solubility in the *γ*‐Al_2_O_3_‐based lattice, with a saturation composition corresponding to Mg/Al ≈ 0.012. Excess Mg therefore forms a separate MgAl_2_O_4_ spinel phase. Consequently, increasing Mg loading above 0.5 wt% primarily increases the relative fraction of the MgAl_2_O_4_ phase, while the chemical identity of each crystallite type remains essentially unchanged. In contrast to Mg, Ga remains uniformly distributed across both crystallite types, with a consistent Ga/Al ratio of approximately 0.0077 (Figures 1c and S4).

All samples exhibit type‐IV N_2_ adsorption–desorption isotherms (Figure S5), indicating predominantly mesoporous structures arising from intercrystalline voids between oxide crystallites. The Brunauer–Emmett–Teller (BET) surface area of the *x*Mg1GaAl samples (Table 1) increases from 217 to 236 m^2^ g^−1^ as the Mg loading increases up to 0.5 wt%, suggesting that Mg incorporation modestly suppresses the growth of the *γ*‐Al_2_O_3_‐type crystallites. Upon further increasing the Mg loading above 0.5 wt%, however, the surface area gradually decreases due to the progressive formation of the MgAl_2_O_4_‐type spinel phase with larger crystallite sizes, as identified by XRD and electron microscopy. Consistent with this trend, 1GaMAO, in which Ga is supported on preformed MgAl_2_O_4_, exhibits the lowest surface area (160 m^2^ g^−1^).

C–H activation over Ga*_x_* Al_2−_ *_x_* O_3_ during PDH is generally considered to occur at tetrahedrally coordinated Ga_IV_ sites [2, 23, 27]. In our recent study, ^71^Ga magic‐angle spinning nuclear magnetic resonance analysis before and after propylene adsorption showed that propylene adsorption predominantly perturbs Ga_IV_ species [29]. This finding supports the use of propylene temperature‐programmed desorption (TPD) to quantify surface‐exposed Ga_IV_ species on Ga*_x_* Al_2−_ *_x_* O_3_, although minor contributions from other catalytically relevant undercoordinated surface Ga environments cannot be excluded [29]. Quantification by propylene TPD (Figures 2a and S6) shows that both the amount of surface Ga_IV_ sites (*Q*(Ga_IV, surf_)) and their fraction relative to the total Ga content (*Q′*(Ga_IV, surf_/Ga_total_)) gradually decrease with increasing Mg loading. This trend can be attributed to the preferential occupation of tetrahedral sites by Mg^2+^ cations [36], thereby driving Ga^3+^ into octahedral positions within the oxide framework. As the Mg loading increases above 2 wt% in the *x*Mg1GaAl samples, the decrease in *Q*(Ga_IV, surf_) and *Q′*(Ga_IV, surf_/Ga_total_) becomes more pronounced not only because of the preferential occupation of tetrahedral sites by Mg^2+^, particularly within the MgAl_2_O_4_‐type phase, but also because of the decrease in surface area associated with the formation of relatively large spinel crystallites (Table 1). In line with this, 1GaMAO exhibits a negligible concentration of Ga_IV, surf_, indicating that Ga^3+^ preferentially occupies octahedral sites in the pure spinel MgAl_2_O_4_ phase.

**FIGURE 2:**
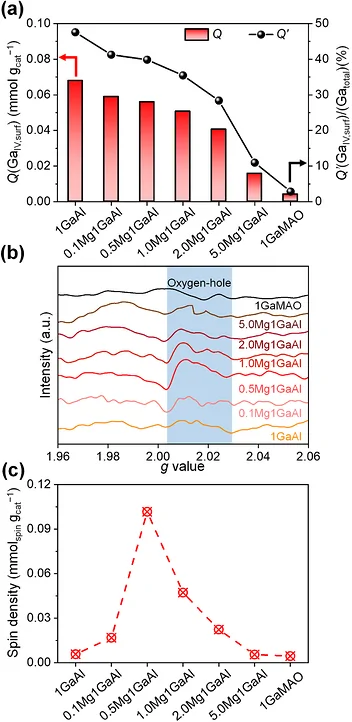
(a) Quantification of surface‐exposed Ga_IV_ sites (*Q*(Ga_IV, surf_)) as a function of Mg loading in *x*Mg1GaAl catalysts. *Q*(Ga_IV, surf_) was determined by propylene TPD, and *Q*′ was defined as *Q*(Ga_IV, surf_) normalized by the total Ga content (Ga_total_) obtained from ICP‐OES. (b) EPR spectra of the prepared catalysts measured at 20 K. The blue‐shaded region corresponds to a signal characteristic of oxygen‐centered hole species. (c) Spin density of EPR‐active oxygen‐centered species normalized by catalyst mass.

Unlike the isovalent incorporation of Ga^3+^, aliovalent substitution of Mg^2+^ for Al^3+^ within the *γ*‐Al_2_O_3_‐type framework at low Mg loadings inevitably introduces charge imbalance, necessitating charge compensation within the lattice. Upon Mg incorporation into the oxide framework, charge compensation can in principle proceed via two pathways: (i) electronic hole generation $\left(\mathrm{MgO}\to {\mathrm{Mg}}_{\mathrm{Al}}^{\prime }+h^{\cdot }+O_{O}^{\times }\right)$, or (ii) oxygen vacancy formation $\left(\mathrm{MgO}\to {\mathrm{Mg}}_{\mathrm{Al}}^{\prime }+0.5V_{O}^{\cdot \cdot }+O_{O}^{\times }\right)$. To identify the dominant charge compensation mechanism in *x*Mg1GaAl samples, electron paramagnetic resonance (EPR) spectroscopy was performed (Figure 2b). Upon Mg loading up to 0.5 wt%, a broad and anisotropic EPR signal at *g* ≈ 2.006–2.030, characteristic of oxygen‐hole species in *γ*‐Al_2_O_3_ [37, 38], progressively increased. Here, “oxygen‐hole species” refers to Mg‐induced lattice oxygen sites with partial O 2*p* electron deficiency and spin density, rather than isolated free‐radical oxygen species. In contrast, no relatively sharp EPR features associated with oxygen vacancies, typically reported at *g* ≈ 2.001–2.005 for F^+^‐type centers in *γ*‐Al_2_O_3_, were detected across the entire Mg loading range, indicating that electronically populated oxygen vacancies do not contribute significantly to charge compensation under the present conditions.

With further Mg addition above 0.5 wt%, however, the signal intensity gradually decreases (Figure 2b). This non‐monotonic behavior can be attributed to the saturation of Mg incorporation within the *γ*‐Al_2_O_3_ lattice at approximately 0.5 wt% Mg, beyond which a distinct MgAl_2_O_4_‐type spinel phase begins to form. In such spinel‐like environments, excess Mg can be accommodated predominantly through cation redistribution within the spinel lattice rather than through electronic hole generation. Consequently, as the fraction of the MgAl_2_O_4_‐type spinel phase increases with excessive Mg loading, the population of EPR‐active oxygen‐hole species correspondingly decreases. These results indicate that maximization of electronically compensated oxygen‐hole species is achieved only when Mg incorporation remains within the solubility limit of the *γ*‐Al_2_O_3_ lattice. Quantitative EPR analysis further confirmed the maximum spin density for 0.5Mg1GaAl (Figure 2c). The apparent spin/Mg ratio of approximately 49% indicates that the EPR‐active oxygen‐centered species are not trace‐level defects but are closely associated with Mg‐induced electronic perturbation of the oxide lattice.

### 2.2 Catalytic Activity and Selectivity in Propane Dehydrogenation

The catalytic properties of the prepared catalysts were evaluated in PDH at 853 K (Figure 3). All catalytic measurements were conducted under differential conditions (propane conversion <10%) to ensure operation in the kinetic regime. No observable change in rate was detected upon varying the total flow rate or particle size, confirming the absence of mass transfer limitations.

**FIGURE 3:**
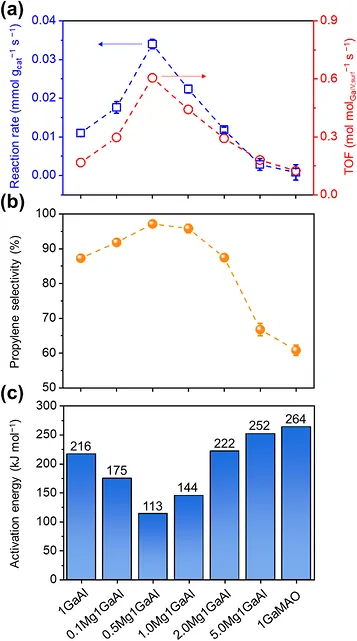
(a) Mass‐specific propylene formation rates and site‐normalized turnover frequencies (TOFs), and (b) propylene selectivity of the prepared catalysts (reaction conditions: 853 K, 20 kPa C_3_H_8_/80 kPa He, WHSV = 35.5–120 h^−1^). Data were collected after 5 min of reaction. (c) Apparent activation energies of the catalysts in PDH.

The mass‐normalized propylene formation rate of Mg‐free 1GaAl was relatively low (0.011 mmol g_cat_^−1^ s^−1^, Figure 3a). It has been previously demonstrated that C–H activation at Ga–O pairs is kinetically facile, whereas recombination of surface hydrogen species to H_2_ constitutes the rate‐determining step on this oxide surface [26, 27, 28, 29]. Upon Mg doping, the catalytic rate exhibited a volcano‐type dependence on Mg loading. The maximum activity (0.034 mmol g_cat_^−1^ s^−1^), approximately three times higher than that of 1GaAl, was observed at 0.5 wt% Mg (0.5Mg1GaAl), after which the rate decreased with further Mg addition (Figure 3a). This remarkable rate enhancement at 0.5 wt% Mg suggests substantial acceleration of the sluggish hydrogen recombination step. When the turnover frequency (TOF) was calculated based on the surface Ga_IV_ sites (Ga_IV, surf_) quantified by propylene TPD, a similar volcano‐type trend was observed. This result indicates that the non‐monotonic variation in mass‐normalized activity cannot be attributed to changes in the number of Ga_IV, surf_ sites induced by Mg doping.

Product selectivity also exhibited a volcano‐type dependence on Mg loading (Figure 3b). Mg‐free 1GaAl showed a relatively low propylene selectivity of 88%, whereas 0.5Mg1GaAl reached the highest selectivity of 97%. The primary byproducts were methane and ethylene (Figure S7), which are typically associated with hydrogen‐mediated C–C bond scission pathways [25]. Therefore, the suppressed formation of these cracking products upon Mg incorporation also indicates that surface hydrogen species are more efficiently removed at the optimal Mg loading. Arrhenius analysis (Figure S8) showed that Mg‐free 1GaAl exhibited a high apparent activation energy (*E*_a_) of 216 kJ mol^−1^ (Figure 3c), comparable to both the reported theoretical barrier for hydrogen recombination on Ga_2_O_3_(100) (201 kJ mol^−1^) and the high *E*_a_ previously observed for gallia–alumina catalysts (197 kJ mol^−1^) [26, 29]. These similarities confirm that hydrogen recombination is the kinetically limiting bottleneck on unpromoted Ga‐based oxide catalysts. Upon Mg incorporation, the *E*_a_ decreased markedly and reached a minimum of 113 kJ mol^−1^ at 0.5 wt% Mg. This reduction approaches the behavior of Pt‐promoted Ga*_x_* Al_2−_ *_x_* O_3_ catalysts with excess Pt loadings, where accelerated hydrogen recombination lowers the apparent activation energy toward the C–H activation regime (∼80 kJ mol^−1^) [29]. Thus, the simultaneous increase in propylene selectivity and decrease in *E*_a_ indicate that optimal Mg incorporation alleviates the bottleneck for hydrogen recombination without requiring Pt.

Notably, the best‐performing composition, 0.5Mg1GaAl, also exhibits the maximum calibrated spin density of EPR‐detectable oxygen‐hole species (Figure 2c). This correlation supports the involvement of Mg‐induced oxygen‐hole species in facilitating hydrogen recombination. Because the spin density measured by EPR represents only the concentration of EPR‐detectable oxygen‐hole species under the measurement conditions, it does not necessarily account for all electronically perturbed oxygen sites, including EPR‐silent or highly delocalized species. Accordingly, the EPR‐active oxygen‐hole species should be viewed as a detectable subset of Mg‐perturbed lattice oxygen sites that may participate in hydrogen recombination. Scheme 1 summarizes the proposed reaction pathway for PDH over Mg‐doped Ga*_x_* Al_2−_ *_x_* O_3_, wherein Mg‐induced oxygen‐hole species facilitate the rate‐determining hydrogen recombination step.

**SCHEME 1:**
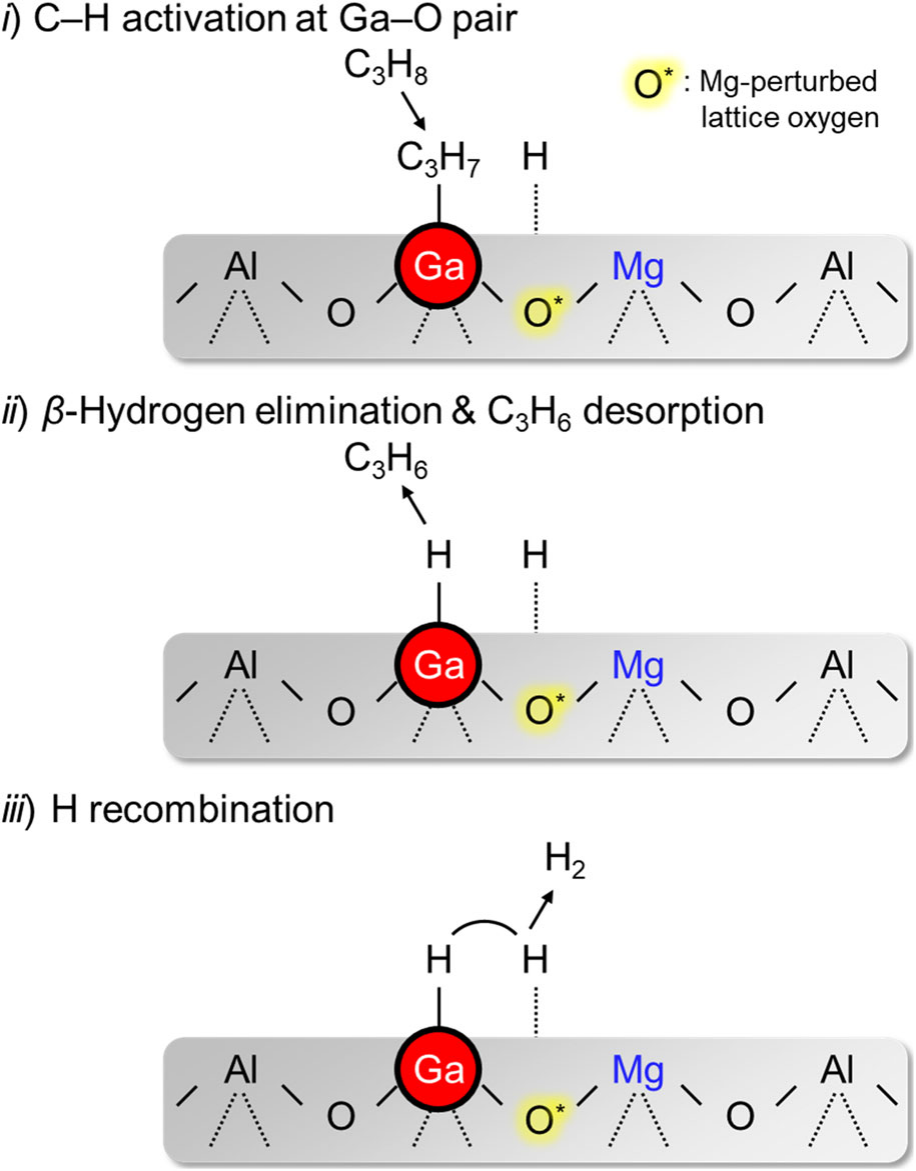
Proposed reaction mechanism for PDH over Mg‐doped Ga*_x_* Al_2−_ *_x_* O_3_ catalysts. O^*^ denotes Mg‐perturbed lattice oxygen with partial O 2*p* electron deficiency and spin density.

### 2.3 Experimental and Theoretical Evidence for Hole‐Assisted Hydrogen Recombination

To experimentally examine the relationship between Mg‐induced oxygen‐hole species and hydrogen recombination kinetics, in situ diffuse reflectance infrared Fourier transform spectroscopy (DRIFTS) experiments were conducted at 773 K. Upon introduction of propane over the 1GaAl and *x*Mg1GaAl catalysts, C–H (2963 cm^−1^) and O–H (3750 cm^−1^) stretching vibrations were observed (Figures 4a and S9), indicating propane chemisorption via C–H bond activation at Ga–O paired sites (Scheme 1, step *i*). In addition, a Ga–H stretching vibration at 1969 cm^−1^ was detected, generated upon *β*‐hydrogen elimination and subsequent C_3_H_6_ desorption (Scheme 1, step *ii*) [29, 39, 40, 41]. In contrast, Ga‐free 0.5 wt% Mg‐doped *γ*‐Al_2_O_3_ (0.5MgAl) exhibited none of these bands, indicating that Mg–O sites alone do not directly mediate C–H activation.

**FIGURE 4:**
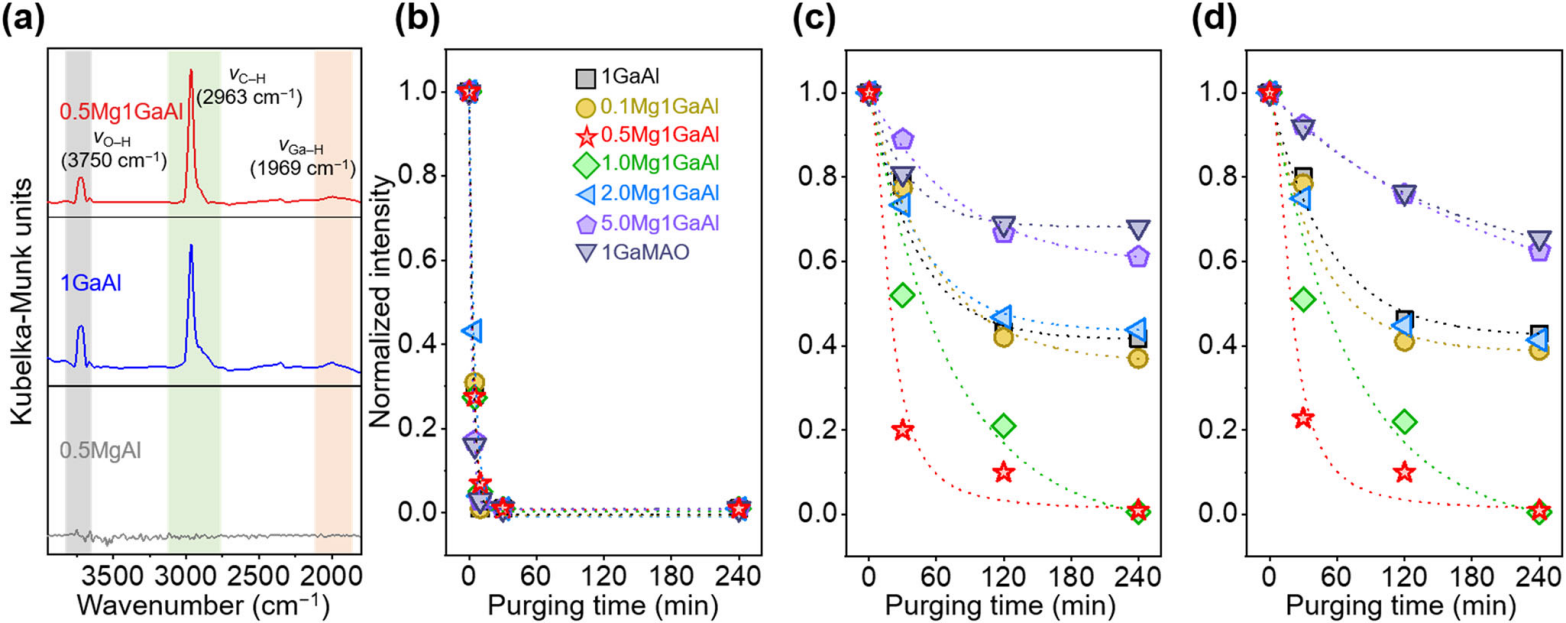
(a) DRIFTS spectra of 0.5Mg1GaAl, 1GaAl, and 0.5MgAl after flowing 20 kPa propane in He for 15 min at 773 K. Normalized band intensities for (b) C–H stretching (2963 cm^−1^), (c) O–H stretching (3750 cm^−1^), and (d) Ga–H stretching (1969 cm^−1^) as a function of time after switching the gas flow to He.

After the propane flow was switched to inert He, the temporal evolution of surface species was monitored. The decay of the C–H stretching band proceeded at similarly rapid rates across 1GaAl and *x*Mg1GaAl samples with different Mg loadings (Figures 4b and S9), suggesting that *β*‐hydrogen elimination of Ga–propyl intermediates and propylene desorption (Scheme 1, step *ii*) remain kinetically facile and largely unaffected by Mg incorporation. In contrast, the intensities of the O–H (Figures 4c and S9) and Ga–H stretching bands (Figures 4d and S9) exhibited substantially slower, Mg‐loading‐dependent decay rates. Notably, the 0.5 wt% Mg catalyst displayed the most rapid depletion of O–H and Ga–H species, whereas both Mg‐free and excessively Mg‐loaded samples showed substantially slower depletion. These results indicate that hydrogen recombination (Scheme 1, step *iii*) is most effectively facilitated at 0.5 wt% Mg loading, consistent with the maximum concentration of oxygen‐hole species identified by EPR analysis. To further corroborate these observations, H_2_–D_2_ exchange experiments were performed (Figure S10). Because HD formation arises from recombination of surface hydrogen species, the HD formation rate serves as a direct measure of hydrogen recombination kinetics [42]. Consistent with the DRIFTS results, the HD formation rate exhibited a volcano‐type dependence on Mg loading, with the 0.5 wt% Mg catalyst showing the highest exchange rate.

To understand the origin of the Mg‐induced oxygen‐hole character and its catalytic role, spin‐polarized density functional theory (DFT) calculations were performed on representative Ga/Mg‐substituted *γ*‐Al_2_O_3_ (001) surface models. Ga and Mg were introduced into neighboring tetrahedral Al sites to construct a representative local Ga–O–Mg motif (Figure S11). This motif was used to examine the local effect of neighboring Mg‐induced electronic perturbation of lattice oxygen, rather than to imply direct experimental observation or exclusive involvement of individual Ga–O–Mg linkages in catalytic cycles.

Oxygen atoms surrounding the dopant pair were classified into four representative environments for direct comparison: shared O, Mg‐side O, Ga‐side O, and distant O (Figure 5a). The spin‐polarized O 2*p* projected density of states (PDOS) reveals that Mg incorporation induces substantial electronic perturbation of neighboring lattice oxygen (Figure 5b). To capture the general effect of Mg substitution rather than a geometry‐specific feature, the O 2*p* PDOS was averaged over four representative Ga/Mg‐substituted configurations (Figure S12). Mg‐side O exhibits the largest unoccupied O 2*p* contribution within 0–1 eV above the Fermi level (0.327), indicating the most pronounced oxygen‐hole character among the examined oxygen environments. Shared O also retains a substantial unoccupied O 2*p* contribution (0.151), whereas distant O (0.114) and Ga‐side O (0.106) exhibit somewhat reduced values in the same energy window. Together, these results indicate that Mg substitution induces an oxygen‐centered electronic perturbation that is strongest in the vicinity of the Mg dopant but extends beyond the immediate Ga–O–Mg motif and gradually attenuates with distance.

**FIGURE 5:**
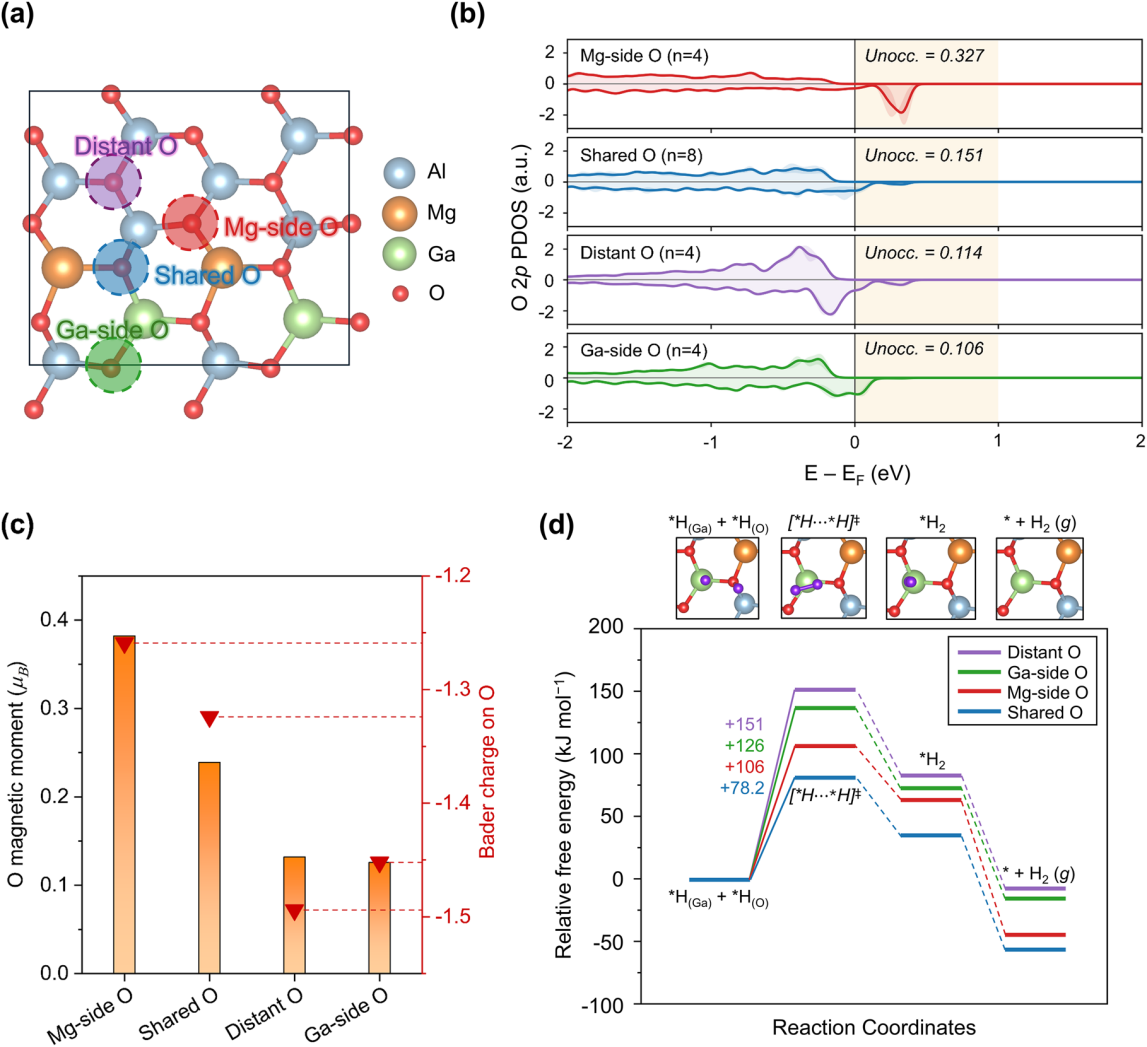
DFT analysis of Ga/Mg‐substituted *γ*‐Al_2_O_3_ (001) surface models. (a) Atomistic model of the most stable Ga/Mg‐substituted *γ*‐Al_2_O_3_ (001) surface and classification of oxygen environments around the Ga–Mg dopant pair. Mg‐side O, shared O, Ga‐side O, and distant O are highlighted. Al, Mg, Ga, and O atoms are shown in light blue, orange, green, and red, respectively. (b) Spin‐polarized O 2*p* PDOS for representative oxygen environments. The shaded region indicates the 0–1 eV energy window above the Fermi level used to integrate the unoccupied O 2*p* contribution. (c) Local magnetic moments and Bader charges of representative oxygen environments in the most stable Ga/Mg‐substituted configuration. (d) Relative free‐energy profiles for H─H coupling and subsequent H_2_ desorption at 853 K, starting from a co‐adsorbed *H_(Ga)_ + *H_(O)_ state. Energies are referenced to the corresponding initial state. Solid horizontal lines denote optimized states, and dashed lines guide the reaction coordinate. Insets show representative structures along the reaction pathway.

Bader charge and local magnetic moment analyses for the most stable Ga/Mg arrangement selected from the initial configurational screening provide a consistent real‐space picture of the oxygen‐hole character (Figure 5c). The Ga and Mg dopants exhibit negligible magnetic moments, indicating that the spin density is not localized on the metal centers. Instead, the largest oxygen‐hole signature appears on Mg‐side O, which exhibits the least negative Bader charge (−1.259) and the largest local magnetic moment (0.381 *μ_B_*). Shared O exhibits intermediate values (−1.324 and 0.239 *μ_B_*), whereas distant O (−1.494 and 0.132 *μ_B_*) and Ga‐side O (−1.452 and 0.126 *μ_B_*) are comparatively more electron‐rich and exhibit smaller magnetic moments. Overall, the Mg‐induced perturbation is localized primarily on lattice oxygen atoms, with the degree of oxygen activation following the order Mg‐side O > shared O > distant O ≈ Ga‐side O.

Additional DFT calculations were performed to examine whether neighboring Mg also facilitates the initial propane C–H activation step. The calculated C–H cleavage barrier slightly increased from 88.8 kJ mol^−1^ on the Mg‐free Ga–O site to 104 kJ mol^−1^ on the Ga–O–Mg site (Figure S13), indicating that Mg incorporation does not promote PDH activity by lowering the initial C–H activation barrier in the present model. This result suggests that the enhanced activity of Mg‐doped Ga*_x_* Al_2−_ *_x_* O_3_ is more directly associated with the subsequent hydrogen recombination step.

We therefore examined how Mg‐induced oxygen‐hole character affects the subsequent hydrogen recombination step at 853 K (Figure 5d). Starting from a co‐adsorbed *H_(Ga)_ + *H_(O)_ state, corresponding to hydrogen atoms bound to Ga and lattice oxygen sites, respectively, the reaction proceeds through H–H coupling to form surface‐bound *H_2_, followed by H_2_ desorption. Among the four examined oxygen environments, shared O exhibits the lowest H–H coupling free‐energy barrier (78.2 kJ mol^−1^), followed by Mg‐side O (106 kJ mol^−1^), Ga‐side O (126 kJ mol^−1^), and distant O (151 kJ mol^−1^). This trend indicates that the most favorable recombination site is not determined solely by the extent of oxygen‐hole character. Although Mg‐side O exhibits the strongest oxygen‐hole character, shared O provides the lowest barrier because the electronically activated oxygen is directly incorporated into the Ga–H/O–H recombination coordinate. By contrast, Ga‐side O still exhibits a relatively high barrier despite its proximity to the Ga center, indicating that geometric proximity to Ga alone is insufficient to promote efficient H_2_ formation. Consequently, the shared oxygen within the Ga–O–Mg motif provides the most favorable environment for H_2_ formation, linking Mg‐induced oxygen‐hole character to the experimentally observed enhancement of hydrogen recombination over Mg‐doped Ga*_x_* Al_2−_ *_x_* O_3_. Although a full description of diffusion‐assisted recombination over all possible surface motifs is beyond the scope of the present study, other Mg‐perturbed oxygen environments may also contribute through surface H migration.

### 2.4 Cyclic Stability Under Dehydrogenation–Regeneration Operation

The stability of the prepared catalysts was evaluated over 30 consecutive dehydrogenation–regeneration cycles. For comparison, industrial benchmark catalysts, K‐promoted PtSn/*γ*‐Al_2_O_3_ and CrO*_x_*/*γ*‐Al_2_O_3_ (denoted as 0.7Pt0.3Sn‐0.8K/Al and 14Cr/Al, respectively, where the numbers indicate metal loadings in wt%), as well as a 0.1 wt% Pt‐promoted 1GaAl catalyst (0.1Pt/1GaAl), were synthesized and tested under identical conditions. Within a single dehydrogenation run, all catalysts exhibited gradual deactivation due to coke formation (Figure S14). Many recent academic studies on PDH catalysts have primarily focused on suppressing catalyst deactivation within a single reaction cycle. However, in industrial PDH processes, complete suppression of catalyst deactivation caused by coke formation is generally difficult to achieve, and the process itself is often designed to utilize the heat released from coke combustion during regeneration. Therefore, a certain extent of catalyst deactivation can be operationally tolerated because coke combustion also contributes to process heat management. From a process perspective, the optimal catalyst may therefore not necessarily be the one exhibiting the slowest deactivation, but rather the one providing the best balance between catalytic activity and regenerability. Accordingly, 30 consecutive dehydrogenation–oxidative regeneration cycles were conducted at 853 K to simulate industrially relevant cyclic operation under severe thermal and oxidative conditions (Figure 6a,b).

The 1GaAl catalyst exhibited relatively low initial catalytic activity (Figure 6a) and propylene selectivity (Figure 6b) owing to its intrinsically sluggish hydrogen recombination capability, while showing only slight deactivation over repeated reaction–regeneration cycles. After 30 cycles, small decreases in surface area, *Q*(Ga_IV, surf_) (Figure 6c), and the HD formation rate, a measure of hydrogen recombination capability (Figure 6d), were observed. These results indicate that the oxide framework remains highly stable, despite a minor loss of accessible active sites and hydrogen recombination capability during prolonged cycling. Meanwhile, 0.1Pt/1GaAl displayed substantially higher initial activity and selectivity, reflecting the enhanced hydrogen recombination capability provided by Pt promotion [27, 28, 29, 30]. However, propane conversion declined rapidly upon repeated cycling. Post‐cycling characterization revealed significant deterioration in hydrogen recombination capability (−86.8%, Figure 6d) due to significant Pt sintering during repeated oxidative regeneration (Figure S15). The changes in surface area and *Q*(Ga_IV, surf_) were only marginal (Figure 6c). These results indicate that deactivation of 0.1Pt/1GaAl upon repeated regeneration originates primarily from the loss of Pt functionality via metal sintering, consistent with earlier studies [28, 29, 30].

**FIGURE 6:**
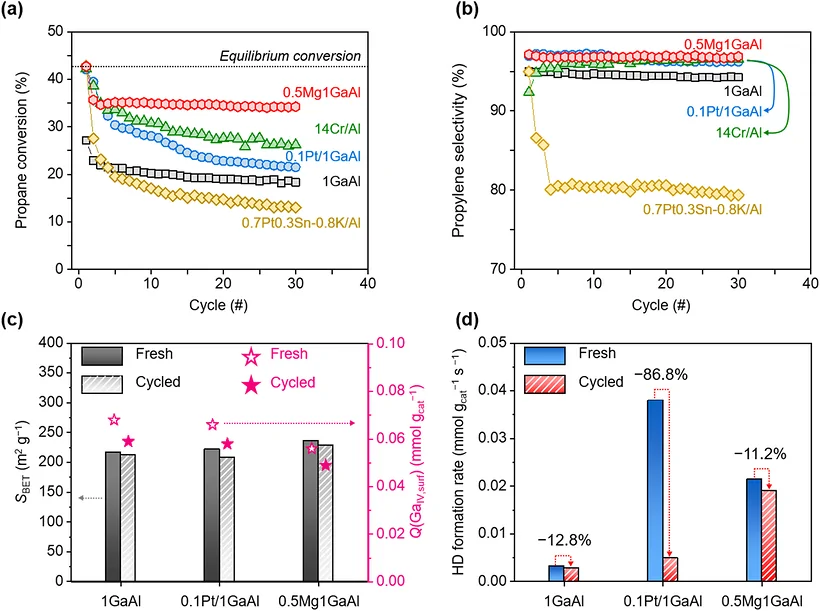
(a) Propane conversion and (b) propylene selectivity of various catalysts in PDH over 30 repeated dehydrogenation–regeneration cycles (dehydrogenation conditions: 853 K, 20 kPa C_3_H_8_/80 kPa He, WHSV = 3.0 h^−1^, 30 min; regeneration conditions: 853 K, synthetic air, 30 min). The reactor was purged with He for 30 min between each half‐cycle. Data were collected after 5 min of reactant gas flow. (c) Comparison of surface area (*S*_BET_) and surface Ga_IV_ sites (*Q*(Ga_IV, surf_)), and (d) HD formation rates during H_2_–D_2_ exchange for both fresh and spent Ga*_x_* Al_2−_ *_x_* O_3_‐based catalysts after 30 reaction–regeneration cycles.

Notably, 0.5Mg1GaAl combined high catalytic activity and selectivity with superior cyclic stability (Figure 6a,b). It underwent only minor deactivation during the first two cycles and then remained relatively stable. Its high catalytic performance can be attributed to Mg‐promoted hydrogen recombination, whereas its resistance to repeated oxidative regeneration arises from its purely oxide framework. To clarify the origin of the slight initial activity loss, we performed additional post‐cycling characterizations. XRD and STEM‐EDS analyses (Figures S16a,b) showed no evidence of MgAl_2_O_4_ formation, while quantitative EPR analysis confirmed that the spin density was largely retained (Figure S16c), indicating preservation of the Mg‐induced electronic perturbation during cycling. In contrast, N_2_ adsorption and propylene TPD revealed small decreases in surface area and *Q*(Ga_IV, surf_) after cycling (Figure 6c), similar to those observed for 1GaAl. These results suggest that the minor activity loss arises primarily from a modest decrease in accessible surface Ga_IV_ sites, likely caused by slight crystallite ripening, while the Mg‐induced electronic promotion of hydrogen recombination remains largely intact.

The industrial benchmark catalysts 0.7Pt0.3Sn‐0.8K/Al and 14Cr/Al were also evaluated under identical cyclic conditions (Figure 6a,b). Both exhibited high initial activity and propylene selectivity during the early cycles. However, 0.7Pt0.3Sn‐0.8K/Al showed rapid performance deterioration upon repeated reaction–regeneration cycling, consistent with the susceptibility of Pt‐based systems to sintering and disruption of the Pt–Sn bimetallic structure under oxidative stress [8, 9]. To mitigate this issue, commercial PtSn processes (Oleflex) often employ controlled oxychlorination to maintain metal dispersion, although such protocols introduce additional operational complexity and materials constraints [1, 2, 9, 15]. The 14Cr/Al catalyst, while oxide‐based, exhibited gradual deactivation over cycling, consistent with diffusion of Cr species into the alumina lattice and the resulting loss of accessible active sites under high‐temperature regeneration [43, 44, 45]. Taken together, these results demonstrate that Mg‐doped Ga*_x_* Al_2−_ *_x_* O_3_ achieves catalytic performance comparable to or exceeding that of Pt‐ and Cr‐based benchmark catalysts, despite containing no precious metals and only 1 wt% Ga as the active component. The ability to deliver high activity, excellent selectivity, and robust cyclic stability within a fully oxide framework highlights lattice‐level electronic engineering as a practical and sustainable strategy for PDH catalyst design.

## 3 Conclusion

In this study, we demonstrated that Mg doping into Ga*_x_* Al_2−_ *_x_* O_3_ enables elimination of the hydrogen recombination bottleneck in propane dehydrogenation without reliance on precious‐metal (e.g., Pt) promotion. Aliovalent substitution of Mg^2+^ into the *γ*‐Al_2_O_3_ lattice generates electronically compensated oxygen‐hole species, as evidenced by EPR spectroscopy. Kinetic analysis, in situ DRIFTS, H_2_–D_2_ exchange measurements, and DFT calculations consistently revealed that these oxygen‐hole species significantly accelerate the rate‐determining hydrogen recombination step, leading to enhanced propylene formation rates and improved selectivity. Importantly, the fully oxide‐based compositions exhibited superior stability over repeated dehydrogenation–regeneration cycles. As a result, Mg‐doped Ga*_x_* Al_2−_ *_x_* O_3_ achieves catalytic performance comparable to or exceeding that of conventional industrial catalysts while containing no precious metals and only minimal Ga loading. These findings establish lattice‐level electronic engineering of oxide frameworks as an effective and practical strategy for simultaneously enhancing intrinsic activity and regeneration stability in PDH catalysts. More broadly, this work highlights the potential of oxygen‐hole‐mediated surface chemistry to overcome kinetic bottlenecks in oxide‐catalyzed dehydrogenation reactions.

## Author Contributions

**DongHwan Oh**: conceptualization, investigation, methodology, validation, writing – original draft. **Jaewoo Jeong**: formal analysis, software, writing – original draft. **Susung Lee**: investigation, validation, formal analysis. **Younghwan Park**: investigation, validation, formal analysis. **Seunghyuck Chi**: investigation, validation, formal analysis. **Yaejun Baik**: investigation, formal analysis, validation. **Hyungjun Kim**: data curation, software. **Hyeyoung Shin**: software, data curation, formal analysis, validation, writing – original draft, writing – review and editing, supervision, methodology. **Minkee Choi**: data curation, supervision, resources, formal analysis, project administration, validation, visualization, writing – review and editing, investigation, funding acquisition, writing – original draft, conceptualization, methodology.

## Conflicts of Interest

The authors declare no conflicts of interest.

## Supporting information



The authors have cited additional references within the Supporting Information [46–50].**Supporting File**: anie73878‐sup‐0001‐SuppMat.docx.

## Data Availability

The data that support the findings of this study are available from the corresponding author upon reasonable request.
