# Supplementary material for: Hole‐Activated Lattice Oxygen Enables Pt‐Free Propane Dehydrogenation by Eliminating the Hydrogen Recombination Bottleneck
Source: Angew Chem Int Ed Engl. 2026 Jul 30;65(40):e1329092. doi: 10.1002/anie.1329092 (PMC13618261; doi:10.1002/anie.1329092)
Supplement: Supplementary file 1 [file ANIE-65-e1329092-s001.docx]

Supporting Information
©Wiley-VCH 2021
69451 Weinheim, Germany

**Hole-Activated Lattice Oxygen Enables Pt-Free Propane Dehydrogenation by Eliminating the Hydrogen Recombination Bottleneck**

DongHwan Oh, Jaewoo Jeong, Susung Lee, Younghwan Park, Seunghyuck Chi, Yaejun Baik, Hyungjun Kim, Hyeyoung Shin,* and Minkee Choi*

**Abstract:** Gallia–alumina (Ga*_x_*Al_2−_*_x_*O_3_) enables efficient propane dehydrogenation (PDH) via C–H activation at Ga–O pairs, but the overall rate is limited by sluggish hydrogen recombination to H_2_. Although Pt promotion alleviates this bottleneck, it introduces drawbacks including precious-metal cost and regeneration-induced sintering. Here we show that Mg doping into Ga*_x_*Al_2−_*_x_*O_3_ electronically activates lattice oxygen to enable rapid H_2_ evolution directly on the oxide, eliminating the need for Pt promotion. Electron paramagnetic resonance spectroscopy reveals the formation of hole-type oxygen species upon Mg incorporation, while kinetic/isotopic measurements and theoretical calculations demonstrate substantially accelerated hydrogen recombination kinetics. In situ infrared spectroscopy further shows rapid depletion of surface hydrogen species, consistent with facilitated H_2_ formation from Ga–H and O–H intermediates. At 853 K, Mg-doped Ga*_x_*Al_2−_*_x_*O_3_ exhibits a threefold higher propylene formation rate than the undoped material at 97% propylene selectivity, outperforming benchmark PtSn/*γ*-Al_2_O_3_ and CrO*_x_*/*γ*-Al_2_O_3_ catalysts. Furthermore, the fully oxide-based catalyst shows the slowest deactivation over 30 dehydrogenation–regeneration cycles. These findings establish lattice-oxygen electronic engineering as a practical strategy for achieving both high activity and exceptional regeneration stability in oxide-catalyzed dehydrogenation chemistry.

DOI: 10.1002/anie.xxxxxxxx

Table of Contents

1. Experimental Section
   1.1. Catalyst Preparation
   1.2. Characterization
   1.3. Computational Details
   1.4. Catalytic Measurements
2. Supporting Figures
3. References

1. Experimental Section

1.1 Catalyst Preparation

1GaAl and *x*Mg1GaAl catalysts were synthesized by the incipient wetness impregnation method. The Mg content was controlled in the range of 0–5 wt%, while the Ga content was fixed at 1 wt%. In a typical synthesis, a controlled amount of Mg(NO_3_)_2_ (Sigma–Aldrich) was dissolved in deionized water, and the solution was added dropwise onto *γ*-Al_2_O_3_ (Strem). The resultant mixture was dried at 373 K and calcined in flowing air at 1073 K for 2 h (ramp rate: 1 K min^−1^). Subsequently, the calcined powder was impregnated with an aqueous Ga(NO_3_)_3_ solution (Sigma–Aldrich) using the incipient wetness method. The resulting mixture was dried and calcined in flowing dry air at 1023 K for 2 h. For the synthesis of 1GaMAO, an aqueous Mg(NO_3_)_2_ solution was impregnated onto *γ*-Al_2_O_3_ to obtain an Mg/Al molar ratio of 0.5, followed by drying at 373 K and calcination in air at 1073 K for 2 h. The resultant MgAl_2_O_4_ was then impregnated with an aqueous Ga(NO_3_)_3_ solution to achieve a Ga loading of 1 wt%. The sample was dried at 373 K and calcined in flowing air at 1023 K for 2 h. The 0.1Pt/1GaAl sample was synthesized by impregnating an aqueous Pt(NH_3_)_4_(NO_3_)_2_ solution (Sigma–Aldrich) onto pre-synthesized 1GaAl, followed by drying at 373 K and calcination in flowing dry air at 1023 K for 2 h.

As commercial benchmark catalysts, 0.7Pt0.3Sn-0.8K/Al and 14Cr/Al were synthesized by the incipient wetness impregnation method. For the synthesis of 0.7Pt0.3Sn-0.8K/Al, an aqueous solution containing H_2_PtCl_6_ (Kojima Chemicals), SnCl_2_ (Sigma–Aldrich), and KNO_3_ (Junsei) was impregnated onto *γ*-Al_2_O_3_ to achieve nominal loadings of 0.7 wt% Pt, 0.3 wt% Sn, and 0.8 wt% K. The 14Cr/Al catalyst was prepared by impregnating an aqueous Cr(NO_3_)_3_ solution (Sigma–Aldrich) onto *γ*-Al_2_O_3_ to achieve a nominal Cr loading of 14 wt%. Both samples were dried at 373 K and calcined in flowing dry air at 1023 K for 2 h.

1.2 Characterization

The elemental compositions of the catalysts were determined by ICP-OES (720, Agilent). Ga 2*p* XPS spectra were recorded using a Nexsa G2 (Thermo Scientific) equipped with a monochromatic Al Kα X-ray source. The binding energies were calibrated against the C 1*s* peak at 284.5 eV. H_2_-TPR experiments were conducted using a BELCAT II (BEL Japan). Typically, 0.3 g of sieved catalyst particles (200–300 μm) were pretreated in He flow (30 cm^3^ min^−1^) at 673 K for 3 h and subsequently cooled to 323 K. Reduction profiles were recorded using a thermal conductivity detector while ramping the temperature from 323 to 1073 K at a rate of 10 K min^−1^ under a flow of diluted H_2_ (5 kPa H_2_/95 kPa He, 30 cm^3^ min^−1^).

XRD patterns were collected using a SmartLab diffractometer (Rigaku) with Cu Kα radiation (40 kV, 200 mA). SEM images were collected using an SU8230 microscope (Hitachi) at an accelerating voltage of 2 kV. HAADF-STEM images and EDS elemental maps were acquired using a Titan Cubed G2 60-300 microscope (FEI) operating at 300 kV. The samples were dispersed in ethanol and drop-cast onto a carbon-coated copper grid. N_2_ adsorption–desorption isotherms were measured at 77 K using a BELSORP-Max (BEL Japan) after vacuum degassing at 673 K for 3 h. The BET surface areas (*S*_BET_) were calculated from the adsorption–desorption isotherms in the relative pressure range of *P*/*P*_0_ = 0.05–0.15, while the total pore volumes (*V*_total_) were determined at *P*/*P*_0_ = 0.95. Pore sizes (*d*_NLDFT_) were determined using nonlocal density functional theory analysis.

Propylene TPD was conducted in a quartz plug-flow reactor (inner diameter: 13 mm) connected to an OmniStar GSD 320 quadrupole mass spectrometer (Pfeiffer Vacuum). Typically, 0.3 g of sieved catalyst particles (200–300 μm) were mixed with 3.0 g of acid-treated quartz sand (200–300 μm) and loaded into the reactor. The mixture was degassed at 673 K for 4 h under He flow (50 cm^3^ min^−1^) and cooled to 323 K. Diluted propylene (10 kPa C_3_H_6_/5 kPa Ar/85 kPa He, 50 cm^3^ min^−1^) was dosed for 30 min, followed by He purging for 1 h at 323 K. Propylene desorption profiles were monitored up to 873 K under a flow of 5 kPa Ar/95 kPa He (50 cm^3^ min^−1^) with a ramp rate of 10 K min^−1^. Propylene concentration was calibrated using the *m*/*z* = 41 signal with Ar as an internal standard, and the desorbed propylene was quantified by integrating the ion current over time.

X-band (~9.65 GHz) continuous-wave EPR spectra were recorded using an EMX spectrometer (Bruker) at 20 K with a liquid helium cryostat. The spectra were acquired with a microwave power of 3 mW, a modulation amplitude of 5 G, a modulation frequency of 100 kHz, and a time constant of 10.24 ms. All samples were pretreated at 673 K for 3 h under He flow prior to measurement. Spin densities were determined by double integration of the baseline-corrected EPR spectra, normalized to the catalyst mass, and calibrated using TEMPO solutions of known concentrations in toluene as external standards. DRIFTS spectra were collected on a Nicolet iS50 spectrometer (Thermo Fisher) equipped with a PIKE cell and a mercury-cadmium-telluride (MCT) detector. Samples were degassed at 773 K for 8 h under He flow prior to measurements. For in situ DRIFTS experiments, propane gas (20 kPa C_3_H_8_/80 kPa He) was introduced into the cell at 773 K for 15 min at a flow rate of 5 cm^3^ min^−1^. The cell was then purged by switching from propane to He at the same gas flow rate. Spectra were recorded every 5 min for 4 h at a resolution of 4 cm^−1^, and 32 scans were averaged.

H_2_–D_2_ isotope exchange experiments were conducted in a quartz plug-flow reactor with an inner diameter of 13 mm, connected to an online quadrupole mass spectrometer (OmniStar GSD 320, Pfeiffer Vacuum). Typically, 10 mg of sieved catalyst particles (200–300 μm) were mixed with 1 g of acid-treated quartz sand (200–300 μm) and loaded into the reactor. The catalysts were pretreated under He flow (50 cm^3^ min^−1^) at 773 K for 1 h and cooled to 323 K. A mixture of H_2_ and D_2_ gas (10 kPa H_2_/10 kPa D_2_/80 kPa Ar, 50 cm^3^ min^−1^) was introduced into the reactor at 323 K. The concentrations of H_2_, D_2_, and HD in the outlet gas were calibrated using the mass spectrometer peaks at *m*/*z* = 2, 4, and 3, respectively. The partial pressure of HD in the outlet stream was always kept below 10% of the equilibrium value to minimize reverse reaction contributions.

1.3 Computational Details

All spin-polarized density functional theory (DFT) calculations^[46]^ were performed using the Vienna Ab initio Simulation Package (VASP).^[47]^ The interaction between core and valence electrons was described using the projector augmented-wave (PAW) method.^[48]^ Exchange–correlation interactions were treated within the generalized gradient approximation (GGA) using the Perdew–Burke–Ernzerhof (PBE) functional.^[49]^ Long-range dispersion interactions were included using Grimme’s DFT-D3 correction.^[50]^ A plane-wave kinetic energy cutoff of 400 eV was used for all calculations. Electronic self-consistency was reached when the total energy change was below 10^−5^ eV. Gaussian smearing with a width of 0.05 eV was employed.

Representative *γ*-Al_2_O_3_ (001) surface slab models were constructed to describe the local oxide environment of gallia–alumina catalysts. Ga and Mg atoms were introduced by substituting neighboring tetrahedral Al sites, generating local Ga–O–Mg motifs that represent Mg-modified Ga–Al oxide ensembles. The slab models were separated by a vacuum layer of 15 Å along the surface-normal direction to minimize spurious interactions between periodic images. Dipole correction was applied along the surface-normal direction. During structural optimization, the bottom two layers of the slab were fixed to mimic the bulk oxide framework, whereas the surface atoms and adsorbates were fully relaxed. The Brillouin zone was sampled using a (4 × 5 × 1) Monkhorst–Pack k-point mesh.

Reaction free energies were evaluated by incorporating zero-point energy (*ZPE*), enthalpy, and entropy ($S$) contributions according to:

$$\begin{aligned} \Delta G= \Delta E+ZPE-T\Delta S \#\left( 1 \right) \end{aligned}$$

where $\Delta E$ denotes the DFT total energy difference.

Hydrogen recombination pathways were evaluated using the climbing-image nudged elastic band (CI-NEB) method. For each oxygen environment, the initial state was defined as a co-adsorbed *H_(Ga)_ + *H_(O)_ configuration generated by hydrogen placement on a Ga–O pair, where hydrogen atoms are bound to Ga and lattice oxygen sites, respectively. The final state was defined as surface-bound *H_2_. NEB images were optimized until the maximum force on each atom was below 0.05 eV Å^−1^. The H–H coupling activation barrier was calculated as

$$\begin{aligned} {\Delta G}_{a}^{H-H}=G_{TS}-G_{IS}\#\left( 2 \right) \end{aligned}$$

where $G_{IS}$ is the free energy of the co-adsorbed *H_(Ga)_ + *H_(O)_ initial state and $G_{TS}$ is the free energy of the highest-energy climbing image along the NEB pathway. The *H_2_ desorption free energy was calculated separately as

$$\begin{aligned} {\Delta G}_{des}^{H_{2}}=G_{surf}+G_{H_{2}}-G_{surf-H_{2}}\#\left( 3 \right) \end{aligned}$$

where $G_{surf-H_{2}}$is the free energy of the surface-bound *H_2_ state, $G_{surf}$ is the free energy of the clean slab, and $G_{H_{2}}$ is the free energy of gas-phase H_2_.

Propane C–H cleavage was evaluated using the CI-NEB method for structurally comparable Ga–O–Mg and Mg-free Ga–O surface models. In both models, the same C–H bond of molecularly adsorbed propane was cleaved. The initial state was defined as adsorbed C_3_H_8_, whereas the final state consisted of surface-bound C_3_H_7_ and a hydrogen atom transferred to the adjacent lattice oxygen. The C–H cleavage activation free energy was calculated as

$$\begin{aligned} {\Delta G}_{a}^{C-H}=G_{TS}-G_{IS}\#\left( 4 \right) \end{aligned}$$

using the same structural constraints and convergence criteria as those employed for the H–H coupling calculations.

1.4 Catalytic Measurements

PDH reactions were carried out in a quartz plug-flow reactor (inner diameter: 9.5 mm). Typically, 20–100 mg of sieved catalyst particles (200–300 μm) were loaded into the reactor and ramped (10 K min^−1^) to 853 K under He flow. After temperature stabilization for 2 h, dilute propane gas (20 kPa C_3_H_8_/80 kPa He) was introduced (150 cm^3^ min^−1^, WHSV = 35.5–120 h^−1^). Products were analyzed by an online gas chromatograph equipped with a GS-Alumina column and a flame ionization detector. Reaction rates and product selectivities were determined at 5 min on stream below 10% of propane conversion. Long-term cyclic stability was evaluated at a WHSV of 3.0 h^−1^ over 30 consecutive dehydrogenation–regeneration cycles. Each cycle consisted of a dehydrogenation step for 30 min (75 cm^3^ min^−1^) and coke oxidation under dry air (100 cm^3^ min^−1^) for 30 min at 853 K. The reactor was purged with He (100 cm^3^ min^−1^) for 30 min between the dehydrogenation and regeneration steps. For 0.7Pt0.3Sn-0.8K/Al, 30 min of H_2_ reduction step was conducted before each dehydrogenation step.

2. Supporting Figures

***Figure S1.*** Ga 2*p* XPS spectra of the Ga-containing catalysts, compared with a *β*-Ga_2_O_3_ reference.

***Figure S2.***  H_2_-TPR profiles of the Ga-containing catalysts and a physical mixture of *β*-Ga_2_O_3_/*γ*-Al_2_O_3_.

***Figure S3.*** SEM images of the *x*Mg1GaAl and 1GaMAO catalysts. The dotted area denotes plate-like crystallites characteristic of the MgAl_2_O_4_-type spinel structure. 1GaMAO exhibited predominantly the plate-like crystal morphology.

***Figure S4.*** HAADF-STEM images and corresponding EDS elemental maps of the Ga-containing catalysts. Elemental compositions were quantified by averaging over five different domains.

***Figure S5.***  N_2_ adsorption–desorption isotherms of the Ga-containing catalysts at 77 K. The isotherms were offset by 150 cm^3^ g^−1^.

***Figure S6.***  Propylene TPD profiles of the Ga-containing catalysts.

***Figure S7.*** Product selectivities for the Ga-containing catalysts in PDH (reaction conditions: 853 K, 20 kPa C_3_H_8_/80 kPa He, WHSV = 35.5–120 h^−1^). The remaining selectivity corresponds to propylene formation.

***Figure S8*.**  Arrhenius plots of the Ga-containing catalysts in PDH.

***Figure S9.*** In situ DRIFTS spectra of the Ga-containing catalysts for (a) C–H stretching (~2963 cm^−1^), (b) O–H stretching (~3750 cm^−1^), and (c) Ga–H stretching (~1969 cm^−1^) after flowing 20 kPa propane in He for 15 min at 773 K, followed by He purging for the specified time intervals.

***Figure S10.***  HD formation rates of the Ga-containing catalysts (reaction conditions: 323 K, 10 kPa H_2_/10 kPa D_2_/80 kPa Ar).

***Figure S11.*** Representative Ga/Mg-substituted *γ*-Al_2_O_3_ (001) surface models considered for DFT calculations. Four distinct configurations (#1–#4) were constructed by substituting Ga and Mg into neighboring tetrahedral Al sites on the *γ*-Al_2_O_3_ (001) surface. Al, O, Mg, and Ga atoms are shown in light blue, red, orange, and green, respectively.

***Figure S12.*** Spin-polarized O 2*p* PDOS of (a) Mg-side O, (b) shared O, (c) distant O, and (d) Ga-side O sites in the four Ga/Mg-substituted *γ*-Al_2_O_3_ (001) surface configurations shown in Figure S11. The vertical line indicates the Fermi level (E_F_), and the shaded region denotes the 0–1 eV energy window above E_F_ used to evaluate the unoccupied O 2*p* contribution associated with oxygen-hole character.

***Figure S13.*** Comparison of propane C–H cleavage at Ga–O sites with and without a neighboring Mg atom on Ga-substituted *γ*-Al_2_O_3_ (001) surface at 853 K. Relative free-energy profiles are shown for the Ga–O–Mg site (red) and the Mg-free Ga–O site (black), together with the optimized initial-state, transition-state, and final-state structures.

***Figure S14.*** (a) Propane conversion and (b) propylene selectivity of various catalysts as a function of time-on-stream in the first cycle of PDH (reaction conditions: 853 K, 20 kPa C_3_H_8_/80 kPa He, WHSV = 3.0 h^−1^).

***Figure S15.*** HAADF-STEM images of the 0.1Pt/1GaAl catalyst (a) before and (b) after 30 dehydrogenation–regeneration cycles (dehydrogenation conditions: 853 K, 20 kPa C_3_H_8_/80 kPa He, WHSV = 3.0 h^−1^, 30 min; regeneration conditions: 853 K, synthetic air, 30 min). The surface-weighted mean Pt particle size was calculated based on the analysis of >50 Pt domains.

***Figure S16.*** (a) XRD patterns for fresh and spent 0.5Mg1GaAl catalysts after 30 repeated dehydrogenation–regeneration cycles. (b) HAADF-STEM image and corresponding EDS elemental maps of the spent 0.5Mg1GaAl catalyst. (c) Calibrated spin densities for fresh and spent 0.5Mg1GaAl catalysts determined from EPR analysis (dehydrogenation conditions: 853 K, 20 kPa C_3_H_8_/80 kPa He, WHSV = 3.0 h^−1^, 30 min; regeneration conditions: 853 K, synthetic air, 30 min).

3. References

[46] W. Kohn, L. J. Sham, “Self-Consistent Equations Including Exchange and Correlation Effects” *Physical Review* **140**, (1965): A1133–A1138. DOI: 10.1103/PhysRev.140.A1133

[47] G. Kresse, J. Furthmüller, “Efficient Iterative Schemes for Ab Initio Total-Energy Calculations Using a Plane-Wave Basis Set” *Physical Review B* **54**, (1996): 11169–11186. DOI: 10.1103/PhysRevB.54.11169

[48] P. E. Blöchl, “Projector Augmented-Wave Method” *Physical Review B* **50**, (1994): 17953–17979. DOI: 10.1103/PhysRevB.50.17953

[49] J. P. Perdew, K. Burke, M. Ernzerhof, “Generalized Gradient Approximation Made Simple” *Physical Review Letters* **77**, (1996): 3865–3868. DOI: 10.1103/PhysRevLett.77.3865

[50] S. Grimme, J. Antony, S. Ehrlich, H. Krieg, “A Consistent and Accurate Ab Initio Parametrization of Density Functional Dispersion Correction (DFT-D) for the 94 Elements H-Pu” *The Journal of Chemical Physics* **132**, (2010): 154104. DOI: 10.1063/1.3382344
